# Gender Bias, Psychological Well-Being, and Professional Adaptation Among Male Nurses: A Scoping Review

**DOI:** 10.3390/healthcare14142095

**Published:** 2026-07-13

**Authors:** Wenjie Zhu, Zhiying Li, Luodi Jiang, Yuerong Han, Yubin Jiang

**Affiliations:** 1 Faculty of Humanities, Management and Science, Universiti Putra Malaysia Sarawak, Bintulu 97008, Malaysia; gs66643@student.upm.edu.my; 2 Department of Nursing, Guangxi Hospital Division of The First Affitiated Hospital, Sun Yat-sen University, Nanning 530022, China; 3 Faculty of Business Administration, University of Macau, Avenida da Universidade, Taipa, Macau 999078, China; yc37050@um.edu.mo (L.J.); yc27013@um.edu.mo (Y.H.); 4 School of Nursing, Luohe Medical College, Luohe 462000, China; jiangyubin713@outlook.com

**Keywords:** male nurses, gender bias, psychological well-being, professional adaptation, scoping review

## Abstract

**Background:** Male nurses form a minority group in many healthcare institutions and can face gender-based stereotypes, patient refusal, gender role assignment, marginalization in the workplace, and questioning of their suitability for working in caring professions. This scoping review examines the literature on gender discrimination, psychological well-being, and adaptation in professional contexts among male nurses and male nursing students. **Methods:** This scoping review was performed according to the PRISMA-ScR guidelines. Searches were performed in Scopus and Web of Science databases and complemented with the use of pre-designed Google Scholar keyword searches. Included studies had a focus on male nurses, men in nursing, or male nursing students and were concerned with gender discrimination, stereotypes, discrimination, psychological well-being, emotional labor, professional identity, burnout, job satisfaction, adaptation, education, organizational support, or retention. Data were extracted and analyzed using narrative and thematic approaches. **Results:** Forty included studies were identified. Four major themes were revealed: (1) gender discrimination and stereotyping; (2) psychological and professional effects; (3) coping strategies and professional adaptation; and (4) organizational responses and support mechanisms. The included studies reported patient refusal, discomfort in intimate care, gendered role assignment, workplace marginalization, and masculinity-related stereotypes. These experiences were discussed in relation to emotional labor, identity conflict, reduced sense of belonging, burnout, job dissatisfaction, work engagement, and turnover intention. **Conclusions:** Gender discrimination against male nurses should be understood as both an interpersonal and organizational issue.

## 1. Introduction

Nursing remains among the most gendered health professions worldwide. According to international workforce statistics, the global nursing workforce increased from 27.9 million in 2018 to 29.8 million in 2023 [1]. Women, however, still make up approximately 85% of the global nursing workforce. Likewise, a gender distribution pattern can be observed in Europe, as 85% of nurses in European Union countries were women in 2022 [2]. While some countries attract a relatively higher proportion of male nurses, e.g., Italy, with male nurses comprising around 23% in 2022, the nursing profession is culturally and organizationally seen as feminine, caring, emotionally expressive, and nurturing [3]. The described trends demonstrate that the underrepresentation of male nurses is not merely an issue of workforce diversity but also a professional and psychosocial matter.

The work of a male nurse is commonly associated with entering an environment where patients, colleagues, educators, and healthcare organizations may assume gendered beliefs about whom a nurse should be [4]. During clinical practice, men may be exposed to stereotypical attitudes toward masculinity and caring, patient rejection, discomfort during the provision of intimate care, doubt about their emotional competence, and expectations about performing physically tough tasks [5]. Such beliefs create a paradoxical image of male nurses as people who are valued for their assumed physical strength, technical competence, or capability to manage crises but are marginalized in providing emotional, intimate, pediatric, obstetric, and relational care [6]. Hence, gender bias toward male nurses should be considered both an interpersonal issue and an organizational phenomenon.

The discussed experiences may have serious implications for the psychological well-being and professional adaptation of male nurses. They tend to monitor their behavior, control emotions, and manage boundaries to avoid misinterpretation of their conduct in situations of sensitive care [7]. Constant exposure to stereotyping, patient rejection, or organizational marginalization may contribute to psychological stress, emotional labor, low professional belongingness, weak professional identity, burnout, job dissatisfaction, and turnover intention [8]. Simultaneously, some male nurses report positive professional experiences, such as pride in nursing, strong relationships with colleagues, career mobility, patients’ appreciation, and resilience in developing a professional identity [9]. Hence, the experiences of male nurses should be viewed through a comprehensive prism, considering barriers, coping resources, adaptive measures, positive experiences, and organizational factors.

Gender discrimination can be understood as a multidirectional social and organizational phenomenon. Within many healthcare and social contexts, women and girls still face extreme forms of gender-based discrimination and violence over the life course. In their review, Di Donna et al. consider gender-based violence during childhood, adulthood, and older age, noting that the COVID-19 pandemic exacerbated several forms of violence and abuse [10]. Existing research indicates that gender inequality affects diverse populations and settings. Meanwhile, this review focuses on another form of gender bias that is rarely discussed in the literature but is important for professional development: stereotypes, discrimination, and psychological burden experienced by men in the traditionally female nursing profession.

Earlier studies and literature reviews have investigated men in nursing from various perspectives, namely, recruitment, retention in the profession, masculinity, education, patient perceptions, and workplace discrimination [11]. Yet, the existing literature remains fragmented as previous studies have focused on a single aspect of the topic. Typically, these studies have concentrated on the reasons for choosing nursing, leaving the profession, negotiating masculinity, or experiences of male nursing students during clinical placements [12]. The combination of gender discrimination with psychological well-being, professional identity, emotional labor, professional adaptation, organizational responses, and career trajectory has received little attention. Moreover, the experiences of male nurses may vary depending on healthcare setting, nursing specialization, career stage, and culture. However, no comprehensive review has ever synthesized such variations in the experiences of male nurses.

Therefore, this scoping review was conducted to map the existing evidence on gender bias, psychological well-being, and professional adaptation of male nurses and male nursing students. Unlike previous reviews that focused on recruitment, retention, and masculinity of male nurses, this review synthesizes evidence on gender stereotyping and discrimination, psychological and professional consequences, coping and adaptation strategies, experiences in education and specialization, positive professional experiences, and organizational responses.

## 2. Methods

### 2.1. Study Design

This study adopted a scoping review design to map the current evidence on gender bias, psychological well-being, and professional adaptation among male nurses. A scoping review was considered appropriate because the topic is interdisciplinary and includes evidence from nursing, psychology, sociology, gender studies, nursing education, and healthcare management. The purpose of this review was not to evaluate the effectiveness of a specific intervention or to pool quantitative effect estimates but to identify the extent, range, and characteristics of the available literature, summarize recurring themes, and identify gaps for future research.

This scoping review was performed and reported according to the Preferred Reporting Items for Systematic Reviews and Meta-Analyses extension for Scoping Reviews (PRISMA-ScR) by Tricco et al. [13], referring to the PRISMA 2020 statement wherever necessary [14]. The completed PRISMA-ScR checklist is provided in the Supplementary Materials. The protocol for this scoping review has not been prospectively registered.

### 2.2. Research Questions

This scoping review was conducted to answer the following research questions:

(1) Which types of gender bias, gender stereotypes, discrimination, and role expectations have been identified by male nurses and male nursing students?
(2) How does the experience of male nurses differ depending on healthcare settings, specialties, career stage, education setting, and cultural background?
(3) What are the professional outcomes associated with gender bias experienced by male nurses? Psychological well-being, emotional labor, professional identity, professional belongingness, job satisfaction, burnout, work–life balance, retention, and turnover intention should be considered.
(4) What coping mechanisms and professional adaptation strategies do male nurses use to deal with gendered expectations, reactions from patients, marginalization at work, and identity negotiations?
(5) What are the positive professional experiences, resources, practices, strategies, and policy solutions that could be helpful for male nurses’ inclusion, professional adaptation, and retention?

### 2.3. Search Strategy and Data Sources

The literature search was conducted in March 2026. Scopus and Web of Science served as the main databases for the literature review. Scopus and Web of Science were chosen for their broad coverage of peer-reviewed research on male nurses, gender issues, and the psychological well-being of healthcare professionals. Google Scholar was used as a supplementary search source to identify additional potentially relevant records to supplement the search in the main databases.

Studies published between 2021 and 2025 were included to identify new trends in male nurses’ experiences of gender biases, discrimination, and associated psychological issues. The keywords were chosen in accordance with this study’s aim and were elaborated throughout preliminary searches. In Scopus, the search was performed via the TITLE-ABS-KEY field, and in the Web of Science Core Collection, via the Topic field. The search was limited to peer-reviewed journal articles published in English between 2021 and 2025.

Keywords concerning male nurses were merged with terms such as gender discrimination, stereotyping, masculinity, work discrimination, emotional labor, mental or psychological well-being, burnout, job satisfaction, professional identity, turnover intention, and career adaptability. The basic concepts included “male nurses,” “men in nursing,” “gender bias,” “gender stereotypes,” “gender discrimination,” “workplace discrimination,” “masculinity,” “mental health,” “psychological well-being,” “psychological distress,” “burnout,” “emotional labor,” “job satisfaction,” “professional identity,” “turnover intention,” and “career adaptation.” Boolean operators were used to combine these terms, and search strategies were tailored to each database’s needs.

Google Scholar was used as an additional search tool but not as the primary database. In Google Scholar, six combinations of the above-mentioned keywords were searched: “male nurses” AND “gender discrimination” AND “professional identity”; “male nurses” AND “gender stereotypes” AND burnout; “men in nursing” AND masculinity AND “professional adaptation”; “male nursing students” AND “clinical placement” AND “discrimination”; “male nurses” AND “patient refusal” AND “intimate care”; and “male nurses” AND “turnover intention” AND “job satisfaction”. In Google Scholar, the first 10 records sorted by relevance were screened for all six combinations. As two searches returned fewer than 10 records, a total of 46 Google Scholar records were screened. Backward and forward citation searching was not performed. The supplementary search was limited to predefined Google Scholar keyword searches.

The full database-specific search strategies for Scopus, Web of Science Core Collection, and Google Scholar are provided in Appendix A.1. Details of the Google Scholar supplementary search procedure, including the exact search strings, sorting method, search date, duplicate handling, and number of records screened for each search, are provided in Appendix A.2.

### 2.4. Eligibility Criteria

Eligible studies included research on male nurses, men in nursing, or male nursing students that was relevant to clinical nursing practice or workforce development in nursing. The articles covered issues related to gender bias, gender stereotypes, gender discrimination, masculinity, workplace marginalization, patient perceptions, psychological well-being, emotional labor, professional identity, burnout, job satisfaction, career adaptation, and retention or turnover intentions. Eligible articles were those published in peer-reviewed journals in the English language and were empirical, theoretical, or conceptual studies that used qualitative, quantitative, or mixed methods or provided review evidence related to the research questions.

Studies were excluded if they focused only on female nurses or nurses in general but did not include a distinct analysis of male nurses, patients, physicians, or other healthcare professionals. Furthermore, studies were excluded if they addressed only clinical technical skills or nursing in general but did not consider issues related to gender bias, psychological well-being, professional identity, professional adaptation, education, retention, or organizational factors among male nurses. Other exclusions included editorials, letters, conference abstracts, dissertations, opinion pieces, or articles that did not have any empirical, conceptual, theoretical, or review contribution. Finally, studies without access to full texts were excluded from this review.

### 2.5. Study Screening

All retrieved records were exported to EndNote 2025 software for organizing references and eliminating duplicates. After duplicates were removed, titles and abstracts were screened using the pre-established eligibility criteria. References that appeared to be eligible and/or potentially eligible were further obtained for full-text screening. Three authors, including the corresponding author, conducted the eligibility screening process.

### 2.6. Data Charting

A data-charting form was prepared to collect important data from all of the papers included in this review. These data included the authors’ names, year of publication, location of publication/country/region, study design, sample, research context, data-gathering tools, theory/conceptual framework, and findings and their relevance to the research questions. The data-charting process was iterative, allowing for flexibility to refine the extraction criteria as knowledge of the field improves via review.

All included studies were sorted according to their primary topic of interest. Particular emphasis was placed on how each of these studies described the manifestation of gender bias, its psychological and professional consequences, the coping strategies employed, negotiations of identity, and the organizational response. Sometimes, full texts were needed to determine the features of each study and its findings.

### 2.7. Data Synthesis

Given the exploratory nature of this scoping review and the heterogeneity of the included articles, the findings were synthesized narratively and thematically. Descriptive approaches were used to synthesize quantitative papers, whereas qualitative, theoretical, and review papers were synthesized thematically. This synthesis did not compute effect sizes or evaluate the effectiveness of interventions; it simply mapped the evidence on the issue and identified recurring themes. Findings from cross-sectional studies were considered associations, not causes, in this literature synthesis.

This synthesis focused on recurring patterns across the included articles rather than pooling individual study findings. Firstly, the included articles were summarized by year of publication, geographical region, study design, sample, and theme. Later, the findings were synthesized according to each research question. The themes that cut across all of these issues were identified to understand how gender bias impacts male nurses’ mental well-being, emotional labor, identity, job satisfaction, and retention. The interactions between individual- and organizational-level factors—such as stigmatization anxiety, emotional containment, and identity conflict—were considered.

### 2.8. Quality Appraisal

A formal risk-of-bias assessment or quality evaluation was not performed since the goal of the current scoping review was not to determine the certainty of evidence or the effectiveness of interventions. The methodological heterogeneity made it difficult to apply a single appraisal tool across qualitative, quantitative, conceptual, theoretical, and review studies.

Despite the absence of methodological quality assessment of individual studies, study characteristics such as design, sample, country/region, methods/instruments or analysis, and relevance to the research questions were extracted during the data-charting process. Hence, this review’s findings should be treated as a thematic evidence map, not as evidence of causal relations or intervention effectiveness.

### 2.9. Ethical Considerations

Ethical approval was not required because this study was based entirely on previously published literature. No human participants were recruited, and no primary data were collected.

## 3. Results

### 3.1. Study Selection

The search identified 616 records, of which 305 were from Scopus, 265 from the Web of Science Core Collection, and 46 from Google Scholar. After removing duplicate papers across all three databases, 246 duplicates were identified; therefore, 370 records remained for title and abstract screening.

During the screening process for titles and abstracts, 319 papers were excluded because they did not meet the inclusion criteria for various reasons: not focusing on male nurses (*n* = 142), involving the wrong population (*n* = 53), not being relevant to the research questions (*n* = 108), and not being substantial in terms of empirical, conceptual, theoretical, or review contribution (*n* = 16). Fifty-one papers were retrieved for full-text screening; however, two papers could not be retrieved in full text, and only forty-nine papers were considered for eligibility.

**Figure 1:**
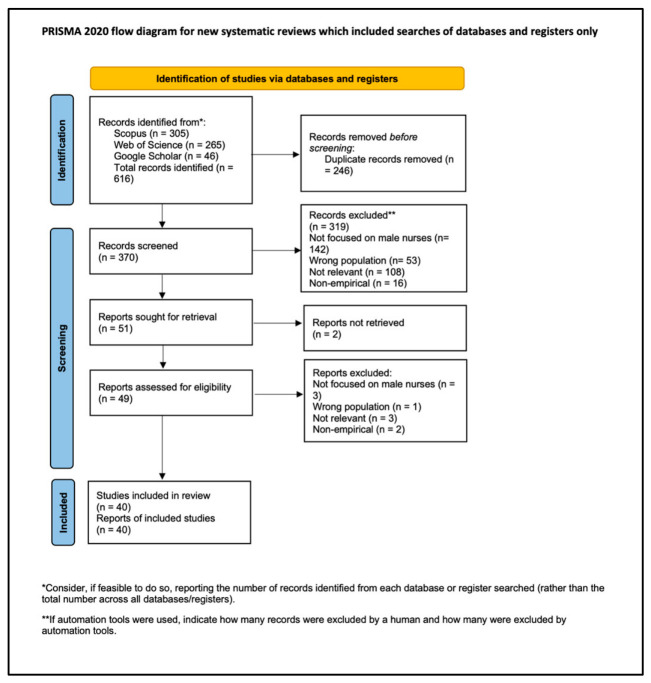
PRISMA 2020 flow diagram of the study selection process, adapted from Page et al. [14]. This work is licensed under a Creative Commons Attribution 4.0 International License (CC BY 4.0). To view a copy of this license, visit https://creativecommons.org/licenses/by/4.0/, accessed on 9 July 2026.

While assessing the full texts of the papers, nine papers were again excluded because they lacked focus on male nurses or male nursing students, were incorrect in terms of population, were not relevant to the research questions, or did not have substantial empirical, conceptual, theoretical, or review contribution. Thus, a total of 40 papers were included in the scoping review synthesis. The study selection process is illustrated in Figure 1.

### 3.2. Characteristics of Included Studies

The studies included in this scoping review were published between 2021 and 2025. The number of earlier articles that served as the basis for defining the theoretical and methodological framework was comparatively minor. The included articles showed a significantly varied geographical distribution, suggesting that the topic of gender-related experiences of men working as nurses has attracted considerable attention across cultures and countries. Asian countries—particularly China and South Korea—contributed most to the research, followed by North America, Europe, Australia, the Middle East, and Africa. The geographic locations of the reviewed articles may have resulted from either regional research priorities or search parameters; thus, they should not be interpreted as indicating that the topic is more significant in one region than in others (Table 1).

Only studies listed in Table 1 were included in the scoping review synthesis. Additional references cited in Section 1 and Section 4 were used for contextual or theoretical background and were not included in the evidence synthesis.

The methodologies used in the included studies were diverse. Quantitative studies were dominant and examined the relationships among perceived gender discrimination, professional identity, emotional labor, job satisfaction, burnout, social support, work engagement, turnover intention, and intention to stay [8,15,16,17,18,19,20,21,22,23,24,25,26,27]. Qualitative studies provided detailed personal accounts from male nurses regarding various issues such as rejection from patients, discomfort associated with intimate care, workplace marginalization, identity construction, and coping mechanisms for dealing with gender-based discrimination [2,5,6,7,9,12,28,29,30,31,32,33,34,35]. Conceptual and review articles offered comprehensive theoretical perspectives on masculinity, the degendering of nursing practice, constructive resistance, recruitment, retention, and the visibility of male nurses [3,4,11,12,36,37].

In terms of research settings, the included studies addressed a variety of hospital settings that are directly applicable to nursing practice, including general hospitals, emergency departments, rehabilitation facilities, pediatrics, psychiatric nursing, labor and delivery, and nursing education [5,17,28,29,31,33,35,37,38,39,40]. Some studies focused on the experiences of male nurses who had already established themselves in clinical settings, whereas others examined male nursing students or newly recruited male nurses [25,28,35,41,42]. In various settings, the experience of being a male nurse was defined by his underrepresentation and the stereotypical views related to care, emotions, technical skills, physicality, and masculinity [3,4,6,12,36].

### 3.3. Thematic Mapping of the Evidence

Four key themes were identified across the included studies: manifestations of gender bias and discrimination, psychological and professional consequences, coping strategies and professional adaptation, and organizational response and support mechanisms. These themes were interconnected since experiences of stereotyping often intersected with psychological stress, professional identity negotiation, work relations, and career continuation decisions.

One major body of evidence concerned manifestations of gender bias, stereotypes, and discrimination toward male nurses. In the literature, male nurses have been regarded as unusual in their profession, perceived as not being nurturing enough, less emotionally expressive, or less naturally suited to providing bedside care [3,4,6,12,36,43]. Stereotypes about male nurses were apparent among both patients and coworkers. For example, male nurses were often appreciated for their physical strength or technical skills but excluded from emotional care, intimacy, or communication-related care [5,6,29,33,36].

Patient resistance toward male nurses was often noted in relation to intimate care, pediatric care, obstetric or labor cases, or other situations involving close physical interaction [14,15,16,18,33,40]. In addition, there were many accounts of patients’ concerns being misinterpreted in their actions during clinical procedures [5,29,30,33]. These concerns appeared to be especially acute in cases where male nurses had cared for female patients or engaged in procedures that involved intimate contact [5,29,33].

The second theme was related to the psychological and professional consequences of gender bias and gender discrimination in nursing. In this regard, male nurses reported experiencing gender-specific anxiety, emotional repression, role conflict, low self-esteem, and identity conflict [22,30,31,37], along with job dissatisfaction, lack of work commitment, engagement, burnout, and intention to leave the profession [8,15,16,18,19,20,21,25,26]. Furthermore, gender discrimination and lack of professional recognition have been associated with problems with belonging and the development of professional identity [3,6,22,24,27].

Coping strategies and professional adaptation were another theme considered in the literature. Coping techniques used by men working as nurses included emotional withdrawal, humor, boundary setting, increased self-monitoring, and redefinition of one’s identity [12,31,32,33]. In some cultures, male nurses used masculine resources, technical skills, decisiveness, or physical strength to gain acceptance from colleagues as nurses [6,32]. In various studies, male nurses’ coping strategies are described as helping them deal with stressors; however, these strategies may reproduce the stereotypes in certain settings [6,12,36].

Organizational response and support mechanisms constituted the last theme addressed in the literature. Some of the measures discussed included mentorship programs, a gender-neutral work environment, an inclusive leadership style, psychological counseling, anti-discrimination training, and public campaigns raising awareness about the issue [11,12,17,18,25,27,34]. In addition, organizational responses to gender-based discrimination in the workplace, which involved reporting mechanisms, handling of sexual discrimination, refusal of treatment, and specific support programs for male nurses, were lacking [7,19,30,33].

### 3.4. Psychological and Professional Outcomes Associated with Gender Bias

Immediate experiences of gender bias that have been previously researched included fear of rejection by patients, fear of misunderstanding, heightened vigilance, and emotional distress [22,29,30,33]. These issues occurred more often while providing intimate care, pediatric care, obstetric services, and medical care for women [5,28,29,31,33,39].

Additionally, gender bias has been defined as an aspect of professional identity and belonging. Male nurses reported defending their career choice, negotiating stereotypes of masculinity, and responding to assumptions about their low predisposition for empathy, caring, and emotional labor [3,4,6,36]. The repetition of such assumptions led to identity conflict, self-efficacy deficiency, emotional detachment, burnout, job dissatisfaction, and turnover intention [6,8,15,16,19,20,21,22,24,26,32].

The professional well-being of male nurses, including work engagement, career identity, job satisfaction, and turnover intention, is frequently mentioned in the reviewed literature. Based on quantitative research, there is an association between gender-related experiences, work engagement, career identity, job satisfaction, organizational support, burnout, and turnover intention [8,15,16,18,19,20,21,25,26,44]. However, while such evidence demonstrates that gender bias could be associated with professional well-being and career development, the cross-sectional design of most of the analyzed studies precludes drawing conclusions about causality and the direction of the relations between the variables.

However, the psychological well-being of male nurses needs to be considered in the context of a setting where gender experiences occur. It is reported that a positive work environment, acceptance among colleagues, and organizational support can prevent problems associated with gender bias. Conversely, poor team culture, lack of recognition, gender separation of tasks, and reporting barriers can lead to psychological and professional strains [11,17,18,25,27,34,35]. Consequently, the psychological well-being of nurses should be understood in terms of workplace, organizational policies, professional practices, and anti-stereotypical approaches to healthcare provision.

### 3.5. Coping Strategies and Professional Adaptation

Strategies used by male nurses in response to gender biases included emotional regulation, emotional distancing, humor, boundary setting, self-monitoring, and effective communication in situations where male nurses felt vulnerable to patient discomfort, rejection, or misinterpretation [12,30,31,33]. In addition, such strategies helped male nurses cope with stress in cases of intimate care, pediatric care, obstetric work, and interactions with female patients. They also required continuous monitoring of their speech, body language, emotional expression, and professional behavior [30,31,33].

Male nurses’ adaptation to gendered expectations may involve identity-based strategies. Male nurses were reported to reinforce their professional identity by demonstrating competence, reliability, ethical behavior, and a patient-oriented approach [9,12,27,32]. Moreover, some studies reported that male nurses used gendered resources, such as physical strength, technical expertise, emotional regulation, and leadership, to obtain acceptance from colleagues and patients [6,32]. On the one hand, such strategies may legitimatize male nurses in certain contexts; on the other hand, they can perpetuate gendered expectations because men will be considered useful in terms of physical, technical, or leadership aspects of nursing only [6,12,36].

Educational experiences have also been associated with professional adaptation. It has been reported that male nursing students face gendered expectations even before entering full professional practice, namely, during clinical practice, rotation in labor and delivery departments, pediatrics, and intimate care [5,28,31,33,35,42]. Such experiences are related to problems of patient acceptance, peer relations, faculty support, and competence in gender-sensitive nursing tasks [25,28,35,42]. Faculty mentoring and visible male role models may be supportive in recruiting people into nursing programs, retaining nurses in the educational process, and the formation of professional identity [5,25,28,31,33].

Professional adaptation is also related to career satisfaction, work–life balance, professional belonging, and long-term career development. Factors such as job satisfaction, work engagement, organizational support, professional identity, and career development possibilities can influence male nurses’ decision to remain in or leave the nursing profession [8,15,16,18,19,20,21,25,26,27]. A positive working environment, peer relations, equitable task allocation, and professional development opportunities can support belongingness and career continuity [11,17,18,25,27,34]. Meanwhile, frequent stereotyping, lack of recognition, a poor workplace culture, and absence of appropriate support in sensitive cases can negatively influence belongingness and increase dissatisfaction and intention to leave [8,19,20,21,26].

Certain organizational contexts also influence male nurses’ adaptation. Workplace culture, policies regarding diversity and inclusion, organizational justice, staffing, leadership style, and professional development possibilities are contextual factors that can influence confidence, belonging, and male nurse retention [11,17,18,25,27,34]. When male nurses are repeatedly assigned physically and technically demanding, security-related tasks and are not recognized for the full scope of their professional activities, stereotypes about the gendered nature of work are reinforced [5,6,29,33,36]. Similarly, limited staffing, policies concerning intimate care, and inappropriate management of patient refusal may create an additional burden on male nurses for navigating gender-related problems [7,29,30,33].

Career stage may be associated with the adaptation process. Nursing students and recently graduated male nurses may be especially vulnerable because they are still developing their professional confidence and clinical identity [25,28,35,42]. They may encounter problems related to patient refusal, gendered expectations, and exclusion during clinical training or early employment. Experienced male nurses may develop more sophisticated strategies for overcoming gender-related problems, varying depending on the clinical context and culture [30,31,32,33,34].

An intersectionality approach may help consider multiple factors that are associated with male nurses’ experiences of gender bias. Age, ethnicity, cultural background, career stage, specialization, and organizational position may play a role [10,11,12,25,27,33]. However, most studies treated male nurses as a relatively homogeneous category and did not consider the intersection of gender with other personal and professional characteristics.

Finally, positive professional experiences must be considered to obtain the full picture. Some studies reported that male nurses experienced pride in nursing, gratitude from patients, collegial relations, career mobility, and resilient professional identity formation [11,12,17,18,25,27,34,38]. These findings suggest that the professional adaptation of male nurses can be analyzed in light of marginalization and coping as well as adaptive resources, a supportive environment, and long-term professional development.

### 3.6. Implications for Organizational Responses and Nursing Workforce Management

The reviewed literature shows that gender discrimination against male nurses is an organizational and nursing workforce management issue. Healthcare organizations have been described as contexts in which gender stereotyping may be reproduced, contested, or normalized through policies, leadership styles, task allocation, incident reporting mechanisms, and professional development opportunities [11,12,17,27,34]. As such, without interventions by healthcare organizations, male nurses have resorted to managing gender issues via informal and individual approaches [7,29,30,33].

Mentorship has often been mentioned as a helpful approach for male nurses [11,12,25,34]. Mentors and male role models have been suggested as helpful tools for fostering professionalism, understanding gendered experiences, and developing strategies for handling difficult situations at work [5,25,28,31,33]. Nonetheless, there is insufficient evidence on what kinds of mentorship are helpful. Hence, mentorship can be considered as a promising practice implication but not as an already proven intervention.

Moreover, inclusive leadership, organizational justice, gender neutrality, and anti-discrimination training can be viewed as potential organizational interventions against gender discrimination [17,18,27,34,35]. They have been discussed as potential approaches for addressing gendered task assignment, harassment, discrimination, patient refusal, and lack of opportunities for professional development. Nevertheless, considering that the existing literature review consists mostly of descriptive, qualitative, cross-sectional, and conceptual studies, these organizational interventions should be regarded as proposed or reported methods.

Lastly, mental health support services receive little attention, although they have been discussed as potentially useful for male nurses who report emotional distress, anxiety, depression, and burnout [22,24,26,30,31]. Since the major outcomes reported are linked to mental state and emotional well-being, organizational measures may be considered to address mental health challenges among male nurses who report repeated exposure to patient refusals and misinterpretations [7,29,30,33].

### 3.7. Evidence Gaps

Despite the recent advancements in the literature dedicated to gender issues, stereotyping, and marginalization experienced by male nurses, various knowledge gaps remain. Most of the included studies had a cross-sectional design; hence, they provided little information about the possible long-term associations between gender bias, the psychological health of male nurses, and their professional identity formation, development, and maintenance [16,19,20,21,22,26].

Little information exists about the possible psychological mechanisms through which gender bias is associated with the professional life of male nurses. Even though there is ample evidence that gender bias is associated with psychological distress, poor professional identity, dissatisfaction at work, and high turnover intention, there have been few attempts to use a psychological theory to explain this phenomenon [15,22,24,25,26,27]. Future research can greatly benefit from applying identity threat theory, self-determination theory, minority stress theory, emotional labor theory, and the job demand–resources model.

Furthermore, few attempts have been made to apply intersectionality theory to study gender bias in the field of nursing. In particular, the most recent studies treated male nurses as a relatively homogeneous group, and little information was collected on their age, nationality, ethnicity, religious beliefs, sexual orientation, specialization, level of experience, and hierarchical position within organizations [11,12,19,25,27,33]. Such factors may significantly affect the experience of gender discrimination and the coping strategies of male nurses.

Very few intervention-focused studies were included in this review. Although mentorship programs, inclusive leadership, anti-discrimination policy, mental health programs, and gender-neutral nursing education have been identified as key potential solutions to gender bias, no information was found on their actual efficacy. Thus, future research should examine whether these interventions are associated with reductions in gender bias, improved psychological well-being, stronger professional identity, and higher retention rates.

Finally, the current literature exhibits significant geographic disparities in distribution. Most of the recently published articles come from Asia, whereas few contributions come from Europe, North America, Africa, etc. More comparative and cross-cultural studies would help identify the specificities of cultural norms, healthcare systems, hierarchical organizational structures, gender ideologies, and other factors that determine the experience of gender discrimination and its consequences for male nurses worldwide.

## 4. Discussion

### 4.1. Principal Interpretation of the Evidence

In terms of the research questions addressed in the selected literature, this scoping review focuses on gender bias, psychological well-being, and professional adaptation among male nurses. The one pattern identified from the literature is that promising approaches to gender discrimination in nursing cannot be limited to the problem of personal attitudes, prejudices, or interpersonal relationships. Rather, gender discrimination implies the structural characteristics of society, where nursing has been associated with femininity and emotions in intimate care provision [3,4,6,12,36,45].

In particular, this review suggests that gender discrimination in nursing emerges in situations where stereotypes become an obstacle to the effective practice of nursing professionals. Gender discrimination may take the form of patients refusing care and experiencing discomfort in intimate interventions. There are also gender-specific divisions of labor in healthcare facilities and certain assumptions about the masculine nature of male nurses [5,29,30,33]. These factors have been associated with repeated stressful situations for male nurses, who must monitor their performance and behavior to avoid having their competence questioned [3,4,6,12,36].

The key implication of this review is that the psychological and professional well-being of male nurses should be addressed in light of both cultural factors and organizational requirements. In other words, stress related to gender bias and stereotypes does not emerge from the presence of gender-specific issues; rather, it arises because male nurses must cope with stereotypical perceptions of their profession to remain in their profession [8,15,16,19,20,21,22,24,26,30].

### 4.2. Gender Bias as a Psychosocial Stress Process

The evidence generated from this scoping review could also be explained in terms of the stress process. Stereotypic perceptions arising from gender expectations affect trust, acceptance, task delegation, and support in delicate clinical interactions. Repeated exposure to such perceptions has been described as being associated with anticipation of stressful situations, which might be associated with hypervigilance, anticipatory anxiety, emotional numbness, and self-threat [5,29,30,33].

Gender discrimination has been associated with mental health outcomes without any assumptions about the cause-and-effect relationship. Most studies under consideration used qualitative, cross-sectional, descriptive, or review approaches that hinder establishing either directionality or causation. Despite this limitation, the findings from the literature suggest a possible stress process: gendered stereotypes may increase psychological demands, encounters necessitating personal care require emotional labor and self-monitoring, and the long-term practice of emotional labor correlates with low identification, low satisfaction, burnout, or turnover [15,22,24,25,26,27,31].

Furthermore, this stress-process model explains why individual coping mechanisms appear insufficient. While emotion detachment, boundary setting, self-monitoring, and identity-shifting strategies may help in dealing with stressful encounters on an individual level, they fail to address the structural origins of gender-related stress. In cases where healthcare organizations have no policies on patient refusals, intimate nursing activities, gender discrimination, or sexual harassment in the workplace, the responsibility for addressing bias falls on individual nurses [7,29,30,33]. Individualization of the problem might protect organizational processes without changing the gendered culture.

Theoretical background allows linking gendered stereotypes and patient refusal to emotional labor, psychological distress, professional identity management, belonging, and retention, as well as the importance of organizational factors, such as mentoring, social support, inclusive leadership, and professional growth opportunities [6,11,17,18,22,24,25,27,30,31,33,34]. It is crucial to consider specialty-specific issues, as professionals’ expectations regarding provision of care may differ depending on the specialization, such as intimate care, pediatrics, obstetrics and labor, psychiatry, and emergency care [5,28,29,31,33,35,37,39].

### 4.3. Professional Identity and the Limits of Masculine Capital

The theme of professional identity became apparent throughout the analysis. The emergence of professional identity among male nurses involves acquiring professional competencies and negotiating the perceived incongruence between masculinity and nursing. Several studies have found that male nurses negotiate this incongruence by emphasizing competence, skillfulness, physical strength, emotional control, or leadership abilities [6,32].

Nevertheless, this approach has limitations. While masculine capital can promote acceptance, it can also be used to reinforce the gendered allocation of nursing labor. If male nurses are respected for physical strength, skillful performance, crisis management, or leadership, their inclusion in the profession becomes possible only if their work involves masculine aspects of nursing care [5,6,29,36]. Hence, features that help male nurses obtain legitimacy may also limit their full professional inclusion, preventing them from fully performing professional duties and feminizing emotional and intimate care.

Regarding gender equity, increasing the number of male nurses does not necessarily lead to greater inclusiveness. Gender-based distribution of roles can co-exist with numerical inclusion if organizations rely on masculine stereotypes. One should focus on ensuring that healthcare institutions accept all aspects of nursing competencies regardless of gender norms to achieve more genuine inclusiveness. Emotional, technical, and relational competencies, as well as leadership skills, are the features inherent to professional nurses.

### 4.4. Implications for Healthcare Organizations and Nursing Management

The findings suggest several potential implications (although careful interpretation is necessary given the lack of evidence from interventions): Addressing gender bias against male nurses must be an essential part of healthcare workers’ psychological well-being and retention plans. In other words, this issue is particularly relevant given current nursing shortages, staff burnout, and employee churn. In cases where gendered experiences cause discontent or attrition among male nurses, their support is needed to ensure both workforce sustainability and diversity [8,19,20,21,26].

Healthcare facilities may consider developing policies that support appropriate and equitable care environments. Policy guidelines for intimate care, patients’ refusal of care based on gender, use of chaperones, patient consent, and protection of health workers could be potential strategies to reduce uncertainty for patients and workers. These policies should strike a balance between patient comfort and gender equity and prevent the reinforcement of gender stereotypes, suggesting that male nurses are inappropriate for intimate and sensitive interactions with patients.

Nursing management should prevent gender segregation at work. Job assignments should be based on skills and expertise levels, training received, workload, and patient needs, rather than presumptions about nurses’ physical abilities or gender appropriateness for certain activities. Moreover, it is the leader’s job to address jokes, microaggressions, discrimination, and exclusion when they occur. The key here is inclusive leadership, which determines whether gender bias will be perceived as a legitimate organizational issue or a matter of personal opinion [17,18,27,34,35].

It is important to implement mentorship programs for male nurses. Mentorship may help male nurses interpret gender-related experiences, build professional self-confidence, and develop strategies for managing sensitive situations in the workplace without avoiding the core tasks of a nurse [11,12,25,34]. Simultaneously, mentorship programs in such situations should focus on preparing men to cope with gender bias and helping organizations improve the workplace environment.

Finally, mental health services should be considered in larger programs of occupational well-being. Gender bias has been related to emotional stress, psychological difficulties, professional burnout, and identity disruption; however, there is no clear information on the effectiveness of interventions to address this issue [22,24,26,30,31]. Accordingly, peer support groups, reflective practice, counseling, and burnout prevention programs may be helpful strategies, although they must be empirically tested among male nurses. Overall, these implications indicate the need for gender-sensitive mentorship, workplace inclusion initiatives, mental health support services, and retention strategies that address both individual adaptation and organizational conditions [5,11,17,18,25,27,28,31,33,34,35].

The reviewed literature suggests that mentorship, inclusive leadership, anti-discrimination training, and psychological support may be promising organizational strategies, but their effectiveness requires further empirical evaluation.

### 4.5. Contributions to the Literature

This review makes a unique contribution to the literature on male nurses by synthesizing research on several related themes. Previous studies have examined topics such as discrimination, recruitment and retention, identity, patient perceptions, and masculine aspects in the nursing profession. By bringing these research topics together, this review identifies how gender discrimination affects the field at the cultural, interpersonal, psychological, and organizational levels, including stereotyping and expectations of who is qualified for care, the impact on interpersonal communication during care encounters, the degree of support available to combat gender discrimination, and the psychological implications thereof.

Another significant contribution of this review is its discussion of the topic with a broader perspective. Previous discussions of gender equity issues usually focus on barriers faced by female medical workers in male-dominated fields and roles. The plight of male nurses highlights the extent to which stereotypical gender norms may affect men when entering professions traditionally associated with women. However, this does not mean that male nurses face the same type of gender discrimination as women working in medicine and healthcare management positions. Rather, it shows that gender norms and expectations narrow the professional landscape of opportunities across all healthcare providers by delegating the tasks of care, emotions, technical skills, and authority to certain gender categories. Lastly, this review contributes to the understanding of gender discrimination in nursing as both a barrier to recruitment and a serious threat to psychological well-being and is potentially useful for workforce management. Healthcare organizations may consider these approaches as potential strategies, but further intervention studies are needed to determine their effectiveness.

### 4.6. Limitations

Several limitations must be considered. First, the selection criteria excluded studies not written in English. This limitation is important because experiences of gender bias, masculinities, nursing work roles, and professional adjustment may vary across different cultural, linguistic, and healthcare contexts.

Second, there were limitations related to the sources used. The literature search included the Scopus and Web of Science databases and was augmented with Google Scholar. While these databases provide access to a large body of peer-reviewed scientific literature, studies available only in discipline-specific databases could have been missed. Google Scholar was utilized as a complementary database; however, only the first 10 entries of each predefined search were evaluated for eligibility. Although Scopus and Web of Science provide broad multidisciplinary coverage, the absence of nursing- and psychology-specific databases such as CINAHL, PubMed/MEDLINE, PsycINFO, and Embase may have limited the comprehensiveness of the search. Therefore, some relevant studies indexed only in these databases may have been missed.

Third, no formal risk-of-bias assessment or methodological quality evaluation was applied. While this approach is in line with the conventions of a scoping review, it means that the findings cannot be viewed as graded evidence or proof of intervention efficacy. The included studies varied in terms of design, sample size, research context, measurements, and methodology.

The heterogeneity of the evidence base is another limitation of this review, which included qualitative, quantitative, conceptual, theoretical, and review studies. Such diversity is suitable for a scoping review; however, it is not possible to directly compare the findings given the qualitative and quantitative evidence presented. Qualitative studies provided context-based descriptions of experiences, processes of identity negotiation, and interaction at the workplace, while quantitative research focused on the associations between experiences of gender bias and mental well-being, job satisfaction, burnout, and intention to leave the current workplace. Therefore, the findings can be interpreted as a thematic map of the evidence but not as aggregated or causative evidence.

Fourth, many empirical studies included in this scoping review had cross-sectional or qualitative designs. These designs provide valuable insights into associations and perceptions; however, they do not show cause-and-effect relationships between gender bias, psychological well-being, professional identity, job satisfaction, burnout, and retention. Many of the most recent studies originated from Asian countries.

### 4.7. Future Research Directions

Future research should investigate the impact of gender discrimination on male nurses’ well-being and development at work. It is essential to conduct longitudinal research to determine whether patients’ rejection, workplace marginalization, or identity threat can predict subsequent burnout, job dissatisfaction, career development, turnover intention, and other related outcomes among male nurses. This line of research will provide evidence that gendered stress accumulates over time and identify moderators and mediators, e.g., professional mentorship, development, and organizational support, that might buffer the negative effects of discrimination.

Research should be based on psychological and organizational theories, including the identity threat theory, minority stress theory, emotional labor theory, self-determination theory, and job demands–resources model. These theories can help researchers better understand how gender stereotypes are internalized and how emotional labor contributes to increased work-related stress and job burnout. They will also explain why a supportive environment at work helps mitigate the effects of gender discrimination.

Researchers should address intersectional issues, as male nurses are a diverse group. Their experiences may be affected by many factors such as age, ethnic origin, sexual orientation, religion, nationality, nursing specialty, career stage, and organizational status. Therefore, comparative research should be conducted, focusing on differences between specialties and cultural groups.

Interventions aiming to reduce the stress experienced by male nurses and promote their professional development need to be thoroughly researched. The recommendations include professional mentorship, leadership and management training, anti-discrimination programs, gender-neutral nursing education, and access to mental health professionals. Currently, little empirical evidence exists regarding the effects of those interventions on male nurses. Thus, this topic should be developed, and mixed-methods research will likely be useful in this context.

## 5. Conclusions

These findings highlight that gender bias and male nurses’ experiences in a female-dominated workplace environment involve a wide range of cultural and organizational constructs associated with stereotypical assumptions regarding gender, nursing, caregiving, emotional expressions, and intimacy with patients. Such bias can take numerous forms, including patient embarrassment, role stereotypes, task segregation, organizational marginalization, and close monitoring of male nurses during sensitive patient care procedures.

Gender bias has been associated with many important psychological and professional challenges for male nurses. Specifically, their experiences have been associated with increased levels of emotional labor, stigmatization, identity issues, lack of belonging at work, dissatisfaction with job responsibilities, burnout, and job turnover. To cope with these problems, male nurses typically utilize various strategies aimed at minimizing gender-based stress, such as emotional distance, boundary management, self-monitoring, identity renegotiation, and utilization of masculinity capital. Although such approaches may help male nurses manage stress in the short term, organizational measures may be needed to provide more sustainable support for gender-bias-related problems.

Future research must go beyond descriptive approaches and move toward longitudinal, theoretically driven, intersectional, and intervention-focused methods that would enable exploring causal associations, evaluating organizational practices for addressing gender bias, and considering factors such as culture, specialty area, experience level, and institutional characteristics that could affect such an issue. Addressing gender bias against male nurses may be important for fostering equity, retention, and sustainability in healthcare organizations.

Implications regarding diversity, retention, and sustained professional adaptation for male nurses emerge from these findings. Future research should adopt longitudinal and interventional study designs comparing different specialties and exploring issues of work–life balance and professional belonging.

## Figures and Tables

**Table 1:** Characteristics of the included studies.

Author/Year	Country/Region	Study Design	Population/Sample	Data Collection/Instruments	Main Relevance to This Review
Padgett [3]	USA	Critical literature review	Literature on men in nursing and diversity	Critical review of diversity and victimization framing	Critiqued how men in nursing are framed in diversity discourse.
Wong et al. [4]	China	Systematic review and meta-synthesis	21 studies; 283 male nurses and 11 male midwives	Meta-synthesis; CASP checklist	Synthesized qualitative evidence on men in nursing and midwifery.
Baker et al. [5]	Australia	Qualitative grounded theory study	23 male nurses and 15 patients	Interviews and observation; grounded theory analysis	Explored male nurses’ and patients’ experiences of gendered care.
Chan and Fang [6]	China	Qualitative study	12 male nurses	Interviews; critical masculinities framework	Analyzed masculinity and identity negotiation in nursing.
Chang and Jeong [7]	South Korea	Qualitative study	10 male nurses	In-depth interviews; thematic analysis	Explored workplace adaptation and gendered experiences.
Kim and Moon [8]	South Korea	Quantitative study	306 male nurses	Secondary analysis of national dataset; regression models	Examined work-related outcomes using national data.
Wu et al. [9]	China	Qualitative interview study	14 male nurses	Semi-structured interviews; Colaizzi’s analysis	Explored gendered workplace experiences and adaptation among male nurses.
Smith et al. [11]	Various	Integrative review	12 studies published between 1987 and 2021	Narrative synthesis; inductive content analysis	Synthesized evidence on men’s experiences in nursing.
Younas et al. [12]	Canada	Qualitative meta-synthesis	16 qualitative studies	Thematic synthesis	Synthesized qualitative findings on men in nursing.
C. Wu et al. [15]	China	Quantitative study	328 male nurses	Validated scales; structural equation modeling and regression	Examined psychosocial factors related to male nurses’ professional outcomes.
Kong et al. [16]	China	Quantitative study	626 male nurses	Latent profile analysis; psychosocial measures	Identified psychosocial profiles related to stress, prejudice, and support.
Kim and Oh [17]	South Korea	Quantitative study	154 male nurses	Practice environment, male nurse social support, and job satisfaction scales	Examined work environment, social support, and job satisfaction.
Lyu et al. [18]	China	Qualitative grounded theory study	25 male nurses	Semi-structured interviews; constructivist grounded theory coding	Developed explanations of male nurses’ adaptation processes.
Chen et al. [19]	China	Quantitative correlational study	627 male nurses	Online questionnaire on turnover intention and related factors	Examined factors associated with turnover intention.
Deng et al. [20]	China	Quantitative descriptive study	13,088 registered male nurses	Online questionnaire; regression analysis	Described demographic and occupational characteristics of male nurses.
Cai et al. [21]	China	Quantitative study	4227 male nurses	Turnover Intention Scale; Decent Work Scale; Colleague Solidarity Scale	Examined decent work, colleague solidarity, and turnover intention.
X. Wu et al. [22]	China	Quantitative study	464 male nurses and male nursing students	Self-esteem, perceived prejudice, and psychological distress scales; PROCESS macro	Examined prejudice, self-esteem, and psychological distress.
Shim and Park [23]	South Korea	Quantitative study	165 male nurses	Questionnaires on gender equity, job satisfaction, esteem, and pride	Examined gender equity perceptions and job-related attitudes.
Mu et al. [24]	China	Quantitative study	Junior male nurses from 10 hospitals	Questionnaires on empathy, emotional intelligence, locomotion mode, and professional identity	Examined factors related to professional identity among junior male nurses.
Yu et al. [25]	Taiwan	Quantitative cross-sectional study	272 newly graduated male nurses	Online questionnaire; structural equation modeling	Examined early-career adaptation among newly graduated male nurses.
Gao et al. [26]	China	Quantitative cross-sectional study	356 male nurses	Measures of burnout, flow, social support, resilience, and task load	Examined burnout and protective psychosocial factors.
Wu et al. [27]	China	Quantitative study	557 male nurses	Measures of career identity, work engagement, and career success	Examined career identity and work engagement.
Iheduru-Anderson and Agomoh [28]	USA	Qualitative study	Male nursing students in labor and delivery rotations	In-depth interviews; thematic analysis	Explored gendered barriers during labor and delivery clinical education.
Lyu et al. [29]	China	Quantitative study	480 male nurses	Structured questionnaires on intention to stay and related factors	Examined factors associated with intention to stay.
Jeong and Chang [30]	South Korea	Quantitative cross-sectional study	155 male nurses	Online survey on workplace sexual harassment and work environment	Examined harassment and work environment among male nurses.
Martínez-Morato et al. [31]	Spain	Qualitative study	12 male nurses in pediatric hospitalization	Semi-structured interviews; qualitative content analysis	Explored male nurses’ experiences in pediatric care.
Mao et al. [32]	China	Descriptive qualitative study	24 male participants	Semi-structured interviews; thematic analysis	Explored men’s experiences and identity in nursing.
Baker et al. [33]	Australia	Qualitative grounded theory study	11 male nurses, 12 care situations, and 15 patients	Interviews, observations, and grounded theory methods	Examined gendered interactions during patient care.
Nerges et al. [34]	USA	Qualitative study	16 male nurses	Video interviews with open-ended questions; thematic analysis	Explored male nurses’ professional experiences and coping.
Shudifat et al. [35]	Jordan	Qualitative study	28 male nursing students	Semi-structured interviews; thematic analysis	Explored male nursing students’ educational and clinical experiences.
Bayuo et al. [36]	Ghana	Conceptual/theoretical paper	Literature on men in nursing, stigma, discrimination, and retention	Literature-based conceptual analysis	Proposed constructive resistance as a retention and thriving strategy.
Reedy et al. [37]	Australia	Scoping review	Studies on men in mental health nursing	Narrative synthesis; PRISMA-ScR checklist	Mapped men’s experiences in mental health nursing.
Keskin et al. [38]	Turkey	Quantitative cross-sectional study	525 nurses	Interpersonal Relationship Styles Scale; Beck Anxiety Inventory; Distress Tolerance Scale	Addressed anxiety, distress tolerance, and interpersonal relations in nursing.
Ageeli and Alharbi [39]	Saudi Arabia	Quantitative study	135 healthcare professionals	Attitudes Toward Men in Nursing Questionnaire	Examined healthcare professionals’ attitudes toward men in nursing.
Gigli et al. [40]	USA	Quantitative study	National sample of registered and pediatric nurses	National Sample Survey of Registered Nurses; statistical analysis	Addressed gender representation and specialty patterns in nursing.
Caputo and Ross [41]	USA	Integrative review	22 articles on male nursing students	Whittemore and Knafl method; MMAT; content analysis	Synthesized male nursing students’ prelicensure education experiences.
Uzun and Tok [42]	Turkey	Quasi-experimental study	24 first-year nursing students	Forum theatre; Gender Attitudes Scale; qualitative opinion form	Evaluated gender-attitude learning through forum theatre.
Abdullah et al. [43]	Malaysia	Qualitative study	14 male nurses and five pak andam professionals	Semi-structured interviews; thematic analysis	Explored gender, care work, and cultural perceptions in Malaysia.
Song et al. [44]	China	Quantitative survey study	394 male nurses	Two-phase survey on stereotypes, exhaustion, resilience, and organizational citizenship behaviors	Examined stereotypes, emotional exhaustion, resilience, and work behaviors.
Dlamini [45]	Swaziland	Historical qualitative study	Male nurse training, 1927–2007	Historical and documentary analysis	Provided historical evidence on male nurse training and gender stereotypes.

## Data Availability

No new data were created or analyzed in this study. All information synthesized in this review is available from the published studies cited in the reference list.

## Supplementary Materials

The following supporting information can be downloaded at https://www.mdpi.com/article/10.3390/healthcare14142095/s1, PRISMA-ScR checklist.

## Appendix A

### Appendix A.1. Database-Specific Search Strategies

**Table A1:** Database-specific search strategies.

Database	Search Field	Search String	Limits/Filters	Records Identified
Scopus	TITLE-ABS-KEY	TITLE-ABS-KEY ((“male nurse *” OR “men in nursing” OR “male nursing student *” OR “male registered nurse *” OR “male student nurse *” OR “man in nursing”) AND (“gender bias” OR “gender stereotype *” OR “gender discrimination” OR “sex discrimination” OR “workplace discrimination” OR prejudice OR stigma * OR masculinity OR “gender role *” OR “patient refusal” OR “patient rejection” OR “intimate care” OR “psychological well-being” OR “psychological wellbeing” OR “mental health” OR “psychological distress” OR anxiety OR stress OR burnout OR “emotional labor” OR “emotional labour” OR “professional identity” OR “professional belonging *” OR belongingness OR “job satisfaction” OR “career satisfaction” OR “work-life balance” OR “work life balance” OR “work engagement” OR “career adaptation” OR “professional adaptation” OR “career development” OR retention OR “turnover intention” OR “intention to stay” OR “clinical placement *” OR “clinical practice” OR mentorship OR mentoring OR recruitment OR “nursing education” OR “organizational support” OR “inclusive leadership” OR “organizational justice” OR “workplace culture” OR “diversity and inclusion”))	Publication years: 2021–2025; Language: English; Document type: article or review	305
Web of Science Core Collection	Topic	TS = ((“male nurse *” OR “men in nursing” OR “male nursing student *” OR “male registered nurse *” OR “male student nurse *” OR “man in nursing”) AND (“gender bias” OR “gender stereotype *” OR “gender discrimination” OR “sex discrimination” OR “workplace discrimination” OR prejudice OR stigma * OR masculinity OR “gender role *” OR “patient refusal” OR “patient rejection” OR “intimate care” OR “psychological well-being” OR “psychological wellbeing” OR “mental health” OR “psychological distress” OR anxiety OR stress OR burnout OR “emotional labor” OR “emotional labour” OR “professional identity” OR “professional belonging *” OR belongingness OR “job satisfaction” OR “career satisfaction” OR “work-life balance” OR “work life balance” OR “work engagement” OR “career adaptation” OR “professional adaptation” OR “career development” OR retention OR “turnover intention” OR “intention to stay” OR “clinical placement *” OR “clinical practice” OR mentorship OR mentoring OR recruitment OR “nursing education” OR “organizational support” OR “inclusive leadership” OR “organizational justice” OR “workplace culture” OR “diversity and inclusion”))	Publication years: 2021–2025; Language: English	265
Google Scholar	Full-text Google Scholar search	Six predefined keyword searches were conducted. The full search strings are shown in Appendix A.2.	Custom range: 2021–2025; Sort by relevance; first records screened for each search	46 records screened

* This is a truncation symbol used to retrieve different word endings and variants of a search term.

### Appendix A.2. Google Scholar Supplementary Search Procedure

Google Scholar was used as a supplementary search source rather than as a primary database. Six predefined keyword searches were conducted using the custom date range 2021–2025. Results were sorted by relevance. For each search, the first 10 records were screened, except when fewer than 10 records were available. A total of 46 Google Scholar records were screened. Duplicate Google Scholar records were removed during the overall deduplication process together with records retrieved from Scopus and Web of Science. Google Scholar records were included in the PRISMA flow diagram and contributed to the total number of identified records.

**Table A2:** Database-specific search strategies.

Search	Search String	Search Date	Records Retrieved	Records Screened	Sorting Method	Notes
Google Scholar 1	“male nurses” “gender discrimination” “professional identity”	March 2026	118	10	Sorted by relevance	First 10 records screened
Google Scholar 2	“male nurses” “gender stereotypes” burnout	March 2026	256	10	Sorted by relevance	First 10 records screened
Google Scholar 3	“men in nursing” masculinity “professional adaptation”	March 2026	5	5	Sorted by relevance	Fewer than 10 records were available
Google Scholar 4	“male nursing students” “clinical placement” discrimination	March 2026	97	10	Sorted by relevance	First 10 records screened
Google Scholar 5	“male nurses” “patient refusal” “intimate care”	March 2026	2	1	Sorted by relevance	Fewer than 10 records were available
Google Scholar 6	“male nurses” “turnover intention” “job satisfaction”	March 2026	1410	10	Sorted by relevance	First 10 records screened
**Total**	—		—	**46**	—	—
